# Estrus Detection in a Dairy Herd Using an Electronic Nose by Direct Sampling on the Perineal Region

**DOI:** 10.3390/vetsci9120688

**Published:** 2022-12-09

**Authors:** Asmaa S. Ali, Joana G. P. Jacinto, Wolf Mϋnchemyer, Andreas Walte, Arcangelo Gentile, Andrea Formigoni, Ludovica M. E. Mammi, Árpád Csaba Bajcsy, Mohamed S. Abdu, Mervat M. Kamel, Abdel Raouf Morsy Ghallab

**Affiliations:** 1 Department of Theriogenology, Faculty of Veterinary Medicine, Cairo University, Giza P.O. Box 12211, Egypt; 2 Department of Veterinary Medical Sciences, University of Bologna, 40126 Ozzano dell’Emilia, BO, Italy; 3 AIRSENSE Analytics GmbH, 19061 Schwerin, Germany; 4 Clinic for Cattle, University of Veterinary Medicine Hannover, Foundation, 30559 Hannover, Germany; 5 Department of Animal Management and Behavior, Faculty of Veterinary Medicine, Cairo University, Giza P.O. Box 12211, Egypt

**Keywords:** cattle, heat detection, MENT-EGAS, pheromones, non-invasive analysis, precision farming

## Abstract

**Simple Summary:**

Fertility a very important field for dairy farms and directly affects their economic success. Therefore, early and accurate estrus detection is crucial, particularly for farms using artificial insemination. During the last decades, several automated sensor-based technologies for estrus detection have been developed. Nevertheless, accurate estrus detection still remains a challenge. In this study, the capacity of electronic nose (EN) technology (MENT-EGAS prototype), which was based on 10 non-specified chemical metal-oxide sensors to detect estrus by the direct sampling of odor from the perineal headspace in Holstein dairy cows, was assessed. Principal component analyses (PCA) were applied and identified high discrimination between proestrus and estrus, and between estrus and metestrus in cycling cows. Additionally, high discrimination amongst estrus in cycling cows and pregnant cows was perceived. Based on these findings, we show for the first time that it is possible to recognize estrus accurately in dairy cattle by direct sampling on the perineal headspace using an EN device during milking. In the future, MENT-EGAS technology could be routinely used on dairy cattle farms as a non-invasive, accurate method for estrus detection.

**Abstract:**

Estrus detection is very important for the profitability of dairy herds. Different automatic systems for estrus detection have been developed over the last decades. Our study aimed to assess the ability of the electronic nose (EN) MENT-EGAS prototype to detect estrus, based on odor release from the perineal headspace in dairy cattle by direct sampling. The study was performed in an Italian dairy farm using 35 multiparous Holstein–Friesian cows. The cows were divided into three groups: group I included 10 lactating 5-month pregnant cows, group II included 19 lactating cycling cows, and group III included 6 cows that were artificially inseminated 18 days before the trial. Odors from the perineal headspace were collected using the MENT-EGAS prototype. In group I, odors were collected once a day for 5 consecutive days. In group II, odors were collected twice daily from day 18 until day 1 of the reproductive cycle. In group III, odors were also collected twice daily from the presumable day 18 of gestation until day 22. Principal component analyses (PCA) of the perineal headspace samples were performed. PCA in group I revealed no significant discrimination. PCA in group II revealed clear discrimination between proestrus and estrus, and between estrus and metestrus but no significant discrimination was obtained between proestrus and metestrus. PCA in group III revealed that in four cows the results were similar to group I and in two cows the results were similar to group II. On day 40 of the presumable pregnancy, the ultrasound examination revealed that only the four cows were pregnant and the other two cows were regularly cycling. On the basis of our findings, we conclude that it is possible to accurately detect estrus in dairy cattle from directly collected odor samples using the MENT-EGAS prototype. This represents the first study of estrus detection using an EN detection by direct sampling. EN technologies, such as MENT-EGAS, could be applied in the future in dairy cattle farms as a precise, non-invasive method for estrus detection.

## 1. Introduction

One of the principal factors affecting the economic success of a farm is reproductive performance [1,2,3]. Several studies reported that fertility problems are one of the most significant causes of culling [4,5]. Thus, early and accurate detection of estrus is crucial. However, proficient estrus detection is an ongoing challenge for efficacious reproductive performance in dairy herds, particularly on farms that resort to artificial insemination (AI) [6]. Even the most fertile sperm or the most skilled inseminator cannot compensate improperly timed AI [7]. 

The detection of estrus on the basis of visual observation is often associated with low efficiency with an estrus detection rate lower than 75% in dairy herds [8]. This fact is often correlated to an increased herd size, reduced estrus duration, and weaker estrus signs due to hormonal synchronization programs [6,9]. Therefore, automated sensor-based technologies have been developed with the aim of mitigating declines in reproductive rates by continuous monitoring and data recording. Some of these technologies, such as pressure-sensitive devices, activity monitoring devices (e.g., pedometer, accelerometer, and video camera), as automated systems of monitoring body temperature and measurement of milk progesterone concentration, are widely used on dairy cattle farms [6]. During the last decade, there has been a clear trend of improvement in estrus detection due to the use of such technologies. However, the accuracy and precision of these devices are still not completely reliable. 

In mammals, the sexual behavior of females and males is induced, and their hormonal status can be modified by stimulation of the vomeronasal organ. The vomeronasal organ has a fundamental role by mediating responses to certain pheromone-like signals [10]. In cattle, a pheromonal function has been suggested for the urine, feces, blood, milk, vaginal fluid, and skin sebaceous glands in the perineal region, playing a role in sexual and social behavior [11,12,13,14,15]. In addition, studies have shown that it is possible to differentiate the odor of urine and vaginal fluid originated from cows having or failing to have estrus [12,13]. In this context, advances in artificial olfaction technology, colloquially called electronic nose (EN), based on odor detection emitted from the perineal area (using cotton swab samples) [7,16,17] from feces [18] and from exhaled breath [19] have been used for estrus detection in cows. However, until date, there are no studies describing estrus detection with a reliable EN technology that could be applied for routine estrus detection in dairy cattle farms. 

A prototype for estrus detection based on a portable EN technology, the so-called “Milking Machine and Electronic Nose Technology-Egypt, Asmaa Shaaban prototype” (MENT-EGAS) has been developed [20,21]. This EN device is based on 10 non-specified chemical metal-oxide sensors. MENT-EGAS uses a pattern generated from the 10 non-specified chemical metal-oxide sensors and various algorithms. In this way, the EN device can recognize up to 10 different compounds or provide a qualitative output based on the vapor composition in the sample and its trained data base, depending on the needs of the user. MENT-EGAS was firstly developed for estrus detection in dairy herds based on the sampling of the perineal odor. In addition, a recent study demonstrated that MENT-EGAS is suitable for accurately recognizing several sources of error, such as environment, feed headspace, and exhaled breath of cows with different diets, proving evidence that this EN device could be used in precision breeding [22]. Our study aimed to evaluate the capacity of the electronic nose (EN) MENT-EGAS prototype for detecting estrus based on odor excretion from the perineal region in Holstein–Friesian dairy cows by direct sampling. 

## 2. Materials and Methods

### 2.1. Instrumentation 

A MENT-EGAS prototype (Patent No. WO2010099800A2) provided by AIRSENSE ANALYTICS GmbH (Schwerin, Germany) was used to detect emanated odor changes from the perineal headspace of the cows. 

**Figure 1:**
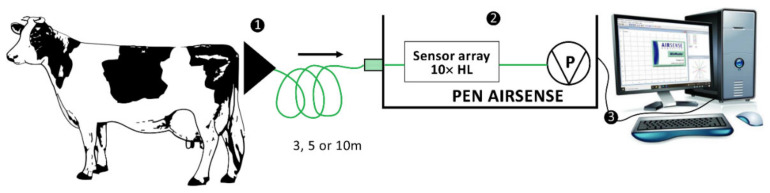
Schematic diagram of the MENT-EGAS prototype system for the sampling of emanated odor changes from perineal headspace of the cows and its three main units: (1) collecting unit, (2) detection, analysis, and identification unit, and (3) result analyzing unit.

The MENT-EGAS prototype consisted of the following units: (1) the collecting unit, (2) the detecting, analyzing, and identification unit, and (3) the result analyzing unit (Figure 1) that were wired connected. 

The collecting unit was represented by a funnel, connected to the second unit using a 2-m-long Teflon tube. The detecting, analyzing, and identification unit was represented by a portable EN with responses of 10 metal-oxide sensors, version 3.5 (PEN 3.5) (Figure S1). The results analyzing unit was represented by the database Winmuster Software, Version 1.6.2.22 Copyright© AIRSENSE ANALYTICS GmbH.

### 2.2. Animals, Housing, and Management 

The study was carried out at the Educational Dairy Farm of the Department of Veterinary Medical Sciences (DIMEVET) of the University of Bologna, Italy, during 5 consecutive days. Thirty-five (n = 35) multiparous Holstein–Friesian dairy cows ranging from 3.5 to 5.5 year’s old were considered in this study. All the included animals were healthy and without a history of reproductive problems. In addition, animals were clinically examined to exclude respiratory, digestive, and metabolic diseases as well as mastitis and lameness. The selected animals were divided into three groups: 

- Group I included 10 lactating, 5-month pregnant cows with an average milk yield of 44 L/day. The animals were selected based on estrus and artificial insemination records and were confirmed to be pregnant by ultrasonographic examination at day 40 and 60 of gestation and just before the beginning of the trial. This group represented the control group as no changes in odors from the perineal headspace were expected [12].

- Group II included 19 regular cycling cows with a reproductive cycle of 21 days with an average milk yield of 58 L/day. The animals were selected based on the estrus data from farm recordings and the estrus expectancy charts. This group represented a study group as changes in the odors from the perineal headspace were expected due to hormonal and pheromonal variations during the reproductive cycle [12].

- Group III included 6 cows that were artificially inseminated 18 days before the trial with an average milk yield of 53 L/day. This group represented a “blind” group as at the moment of the trial there was no confirmation of pregnancy. The animals were selected based on AI records. Four of them were confirmed to be pregnant at day 40 of gestation by transrectal ultrasonographic examination. Two cows were confirmed to be cycling at the time of the first pregnancy diagnosis. 

The animals of all groups were housed in the same free-stall system in a semi-closed and well-ventilated barn with curtained sidewalls and had similar management (capacity, ventilation, housing type, watering, feeding, and manure cleaning up). They were milked two times a day at 5:30 am and 4:30 pm. 

### 2.3. Sampling and Measurement of Milk Parlour Environment

Before starting the study, measurements from the milking parlor environment were collected and analyzed as described in Ali et al. 2022 [22] to avoid possible error sources potentially influencing the EN measurements of odors from the perineal headspace. 

In addition, every time before starting sampling and measurement of odors from the perineal headspace from the cows, measurements from the milking parlor environment were collected, analyzed, and compared with PCA to the initial one to check if there might be a different sensor response which could be detected. 

### 2.4. Sampling and Measurement of Odors from the Perineal Headspace 

Odor samples from the perineal headspace were obtained for each cow during milking by placing the collecting unit of the MENT-EGAS prototype 10 cm from the perineal region (direct sampling).

In group I, odors were sampled from the perineal headspace once a day (during morning milking) for 5 consecutive days. 

**Figure 2:**
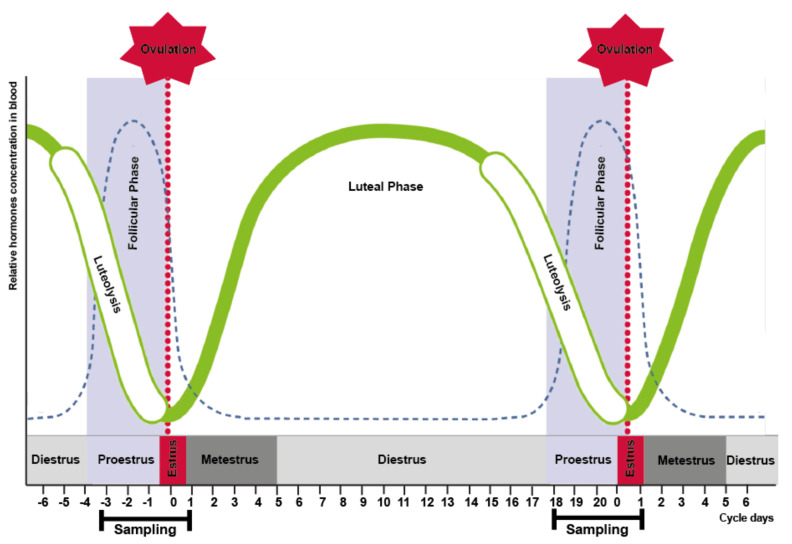
Sampling days according to phases of the bovine estrus cycle. Sampling started on day 18 of the reproductive cycle and finished at the day 1 of the following cycle.

In group II, odors from the perineal headspace were sampled twice daily (during morning and evening milking) from day 18 until day 1 of the reproductive cycle (Figure 2). 

In group III, odors from the perineal headspace were sampled twice daily (during morning and evening milking) from the day 18 of presumed gestation until day 22. 

The sampling of the odors from the perineal headspace was always performed by the same person and EN device to reduce variations and control extra features that can be associated with measurement mistakes. Two repeated measurements were obtained for each sample. 

### 2.5. Response of the Sensor and Data Analysis 

Each measurement was obtained in 50 s followed by a cleaning phase of 60 s. For each measurement, vectors at 47 and 48 s at were acquired and appended to determine a pattern for further analysis by principal component analysis (PCA).

PCA reduces the dimensionality of such dataset sets, thereby increasing interpretability while minimizing information loss. This is accomplished by setting up new non-correlated variables that consecutively maximize the variance. The search for these variables, so-called principal components, is reduced to solve a value/vector problem, and consequently these variables are set out by the dataset, which makes PCA an adaptive data analysis method. By applying PCA, if there is discrimination, it will be possible to recognize the discrimination power between the different samples. PCA was used as a preliminary comparison of perineal headspace samples between group I, group II, and group III. The PCA technique was performed to decrease the dimensionality of complex obtained datasets (data from a ten-dimensional room due to the ten used sensors) into smaller dimensions to maximize the variance, increase the interpretability, but at the same time minimize information loss. Data transformation was performed and graphical plots were obtained [7].

### 2.6. Ethics Statement

Herein, an institutional or official ethical approval was not required because exclusively non-invasive techniques were used. All cows were inspected with the consent of the owner and handled according to good ethical standards.

## 3. Results

### 3.1. Odors from Milk Parlour Environment

No significant difference was detected between the initial measurements from the milking parlor environment and the measurements performed every time before starting the sampling and measurement of odors from the perineal headspace from the cows. In addition, MENT-EGAS was not affected by humidity and temperature.

### 3.2. Odors from the Perineal Headspace in Group I

The 10 metal-oxide sensors of MENT-EGAS could respond for direct sampling but no significant differences in the odor were detected. 

**Figure 3:**
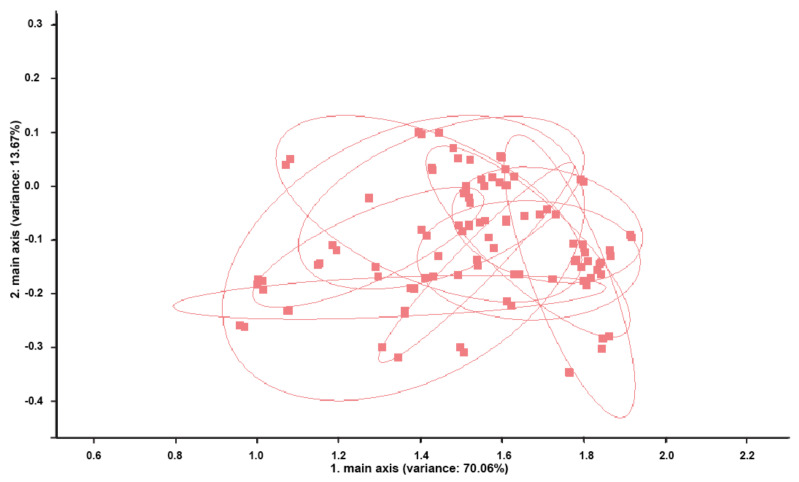
Principal components analysis (PCA) of perineal headspace sample measurements of the 10 cows from group I. Measurements of the perineal headspace samples show no discrimination between cows (all blots are overlapping). Each blot represents a single cow. The percentages of the data matrix described by the relevant components and function is indicated in parenthesis.

PCA of the perineal headspace samples revealed no significant discrimination in the samples collected in five consecutive days during pregnancy (Figure 3). 

### 3.3. Odors from the Perineal Headspace in Group II

**Figure 4:**
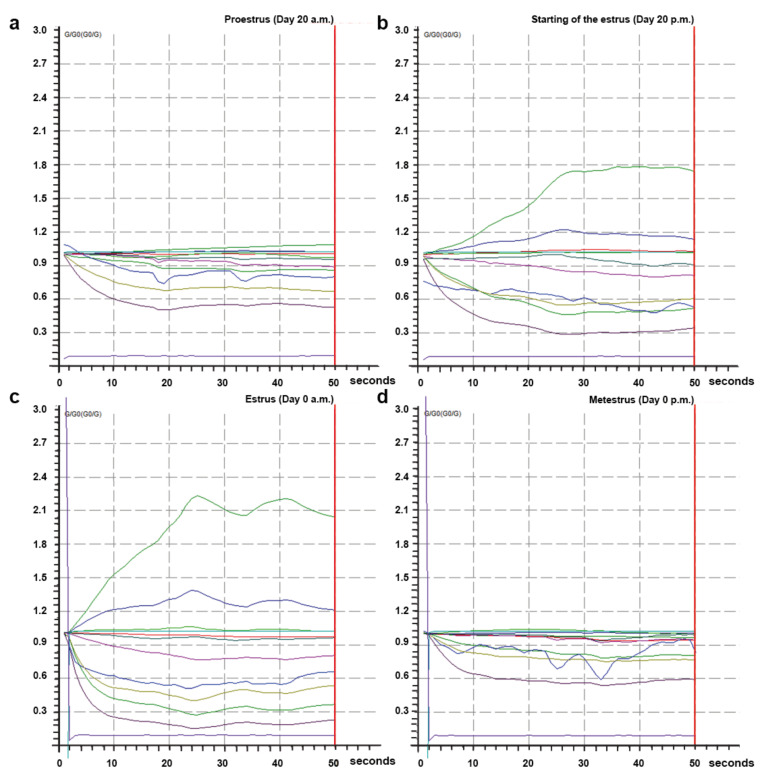
Response from the 10 metal-oxide sensors at different phases of the reproductive cycle to perineal odor in a cow from group II. (**a**) Response from the 10 metal-oxide sensors at proestrus (day 20, a.m.). (**b**) Response from the 10 metal-oxide sensors at the beginning of the estrus (day 20, p.m.). (**c**) Response from the 10 metal-oxide sensors at estrus (day 0, a.m.). (**d**) Response from the 10 metal-oxide sensors at metestrus (day 0, p.m.). The x-axis represents detection time, and the y-axis represented the changes of G/G0 values of EN sensors. Each colored line represents one sensor.

The 10 metal-oxide sensors of MENT-EGAS could respond to direct sampling and detect odor changes in the perineal headspace samples between proestrus and estrus and between estrus and metestrus (Figure 4). The sensor resistance to the odors during estrus (Figure 4b,c) was higher when compared with the proestrus (Figure 4a) and metestrus (Figure 4d). 

**Figure 5:**
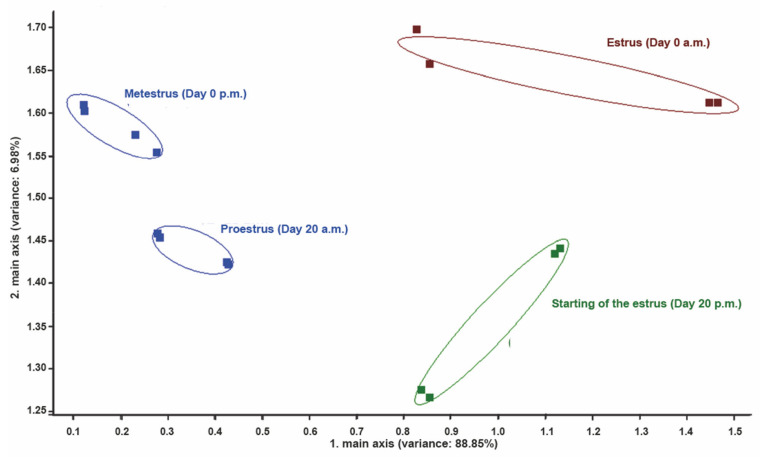
PCA of perineal headspace samples measurements at different phases of the reproductive cycle in a cow from group II. Note the low discrimination between proestrus (day 20 a.m.) and metestrus (from day 0 p.m.). Note the high discrimination between proestrus (day 20 a.m.), beginning of estrus (day 20 p.m.) and most intensive estrus (day 0 a.m.), and between beginning of estrus (day 20 p.m.), most intensive estrus (day 0 a.m.) and metestrus (from day 0 p.m.). Numbers in parentheses indicate percentages of the data matrix described by the relevant components and functions.

PCA of perineal headspace samples revealed low discrimination between proestrus (from day 18 a.m. until day 20 a.m.) and metestrus (from day 0 p.m. until day 1 p.m.); clear discrimination between proestrus (from day 18 a.m. until day 20 a.m.), beginning of estrus (day 20 p.m.), and most intensive estrus (day 0 a.m.); clear discrimination between beginning of estrus (day 20 p.m.), most intensive estrus (day 0 a.m.) and metestrus (from day 0 p.m. until day 1) (Figure 5). 

### 3.4. Comparison of the Odors from the Perineal Headspace between Group I and Group II

**Figure 6:**
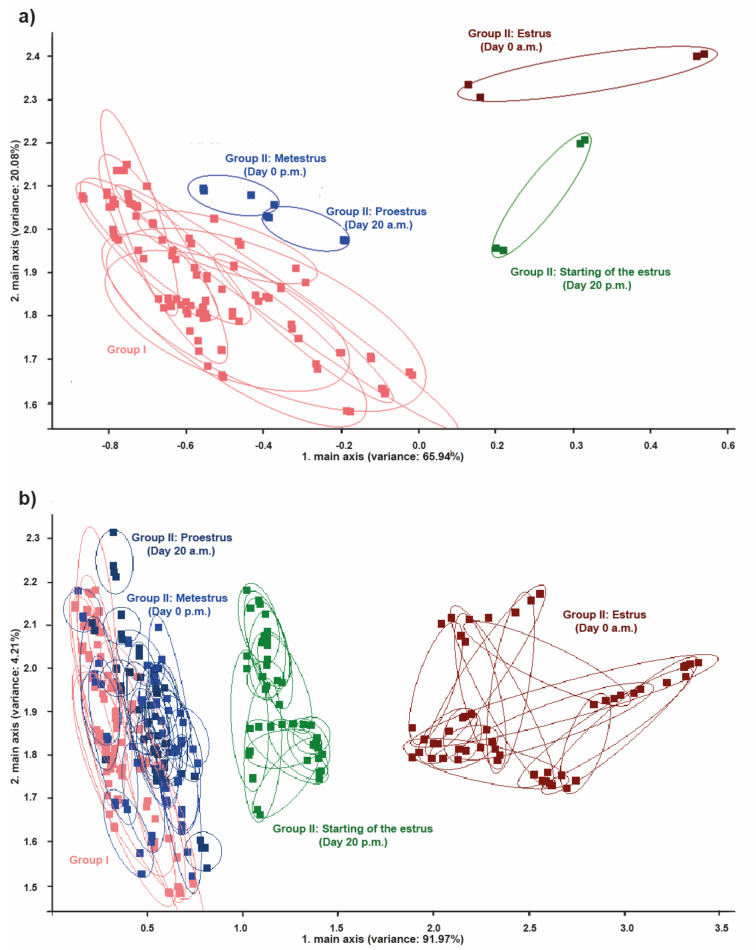
PCA between groups I and II. (**a**) Comparison of PCA between the 10 cows from group I and a cow from group II and (**b**) comparison of PCA between the 10 cows from group I and the 15 cows from group II. Note the low discrimination between the pregnant cows from group I and the regular cycling cow from group II in proestrus (day 20 a.m.) and metestrus (from day 0 p.m.). Note the high discrimination between the pregnant cows from group I and the regular cycling cow from group II in estrus (day 20 p.m. and day 0 a.m.).

By comparing the PCA obtained results, no significant discrimination was present between the pregnant cows from group I and the cycling cows from group II in metestrus and proestrus. However, high discrimination was noticed between pregnant cows from group I and cows in estrus from group II (Figure 6a,b). Using PCA, three clusters were noticed after comparison of the odors from the perineal headspace between group I and group II. The first cluster included the cows from group I and the cows from group II in proestrus and metestrus (Figure 6b). The second cluster included cows from group II in the starting of the estrus and the third cluster included cows from group II in the maximum of estrus (Figure 6b). 

### 3.5. Odors from the Perineal Headspace in Group III

The 10 metal-oxide sensors of MENT-EGAS could respond for direct sampling and detect odor changes in the perineal headspace samples in two, presumably pregnant cows. The results obtained for these two animals resembled the results from group II. In fact, at the presumable day 40 of pregnancy, transrectal ultrasonography revealed that the cows were regularly cycling. For the remaining four cows, the results resembled the results from group I. For these four animals, the pregnancy was confirmed at day 40 of gestation using ultrasound examination. 

## 4. Discussion

Achievement of maximal sensitivity for estrus detection is crucial for the success of programs that use automated estrus detection systems as the only or principal method for identifying suitable cows for insemination. Herein, the MENT-EGAS prototype was capable to respond to direct sampling on the perineal region with a non-invasive method and to accurately detect estrus in Holstein–Friesian dairy cows during milking. 

PCA analyses demonstrated no significant discriminations in the samples collected for five consecutive days within group I (pregnant cows). In addition, no significant discrimination between proestrus and metestrus in group II (cycling cows) was perceived. Similarly, when comparing the PCA results of the pregnant cows from group I and those of the cycling cows from group II, no significant discrimination was found between them during metestrus and proestrus. On the contrary, PCA analysis demonstrated in group II (cycling cows) significant discriminations between proestrus and estrus and between estrus and metestrus. Likewise, high discrimination was noticed between pregnant cows from group I and cows in estrus from group II. Altogether, these findings showed that the EN device used in this study is capable to accurately detect estrus in dairy cows. Our results are comparable with previous studies that used different EN devices for estrus detection from samples of the perineal region. In these previous studies, the samples were collected as disposable swabs from the perineal region [7,16]. In our study, the samples were obtained by direct sampling without the cows being touched, representing a non-invasive method for estrus detection. 

Using the pattern generated from the 10 non-specified chemical metal-oxide sensors of MENT-EGAS and various algorithms, it was possible to provide a direct answer “estrus” or “non-estrus”. Even though the identification of specific components was not performed, there was a clear difference in sensor response between proestrus and estrus and between estrus and metestrus (Figure 4), demonstrating the proposed EN device accuracy. In fact, it is known that estrus behavior in cows is linked with several volatile compounds such as amines, ethers, diols, alcohols, and ketones, with greater concentration of methylheptanol and acetaldehyde, and some of them are suggested to act as pheromones [12]. Several body secretions such as urine, feces, milk, sweat, and vaginal fluid have been suggested to have a pheromonal function that plays a role in sexual and social behavior in the bovine [11,12,13,14,15]. In particular, one study showed that acetic acid, propionic acid, and trimethylamine in vaginal fluid appear only during estrus but not during any other phases of the reproductive cycle, suggesting that vaginal fluid is the principal source of pheromone production during estrus and, therefore, represents the more efficient body secretion for estrus detection in cattle [13]. In contrary, feces produce other volatile organic compounds that are not estrus-related, which might interfere with the sensor response of EN’s during the measurements [22,23,24]. In cattle, the perineal region is frequently soiled with feces and vaginal fluid during estrus. Nonetheless, MENT-EGAS was able to distinguish estrus from non-estrus phases, even with the presence of possible error sources such as fecal contamination.

The EN-based MENT-EGAS prototype showed an interesting potential application in the early detection of repeat breeders as demonstrated by the results obtained within group III, where it was possible to detect changes in the perineal headspace samples resembling estrus 21 days after AI in two cows presumed pregnant. Repeat breeder cows represent an important economic loss source in the dairy industry, which is directly associated with the increased number of inseminations and associated costs, the increased number of days open, and consequently lower milk production [25]. The cause for this condition seems to be very complex and multifactorial [26,27,28]. In spite of the capability to detect repeat breeder cows, it is crucial to apply an efficient therapy and proper herd management, otherwise repeat breeder syndrome remains largely uncontrollable. 

Furthermore, through using a MENT-EGAS prototype for estrus detection, it is possible to prevent false positive and/or false negative results that can occur when estrus detection is based on behavioral signs of estrus, such as primary and secondary signs, including activity and social interactions. Considering the primary signs of estrus, “standing to be mounted” is considered being the furthermost distinctive visual sign for identifying estrus in cattle [6]. Unfortunately, a decrease in this behavior has been observed mostly in high-yielding cows [29]. This phenomenon might be the consequence of the reduction in estradiol concentration in serum because of the increased rate of hepatic metabolic clearance of steroid hormones [30,31]. “Mounting behavior” is a secondary sign of estrus, and it seems to be more precise than standing behavior [32]. However, the number of mounts per cow depends largely in housing conditions [33]. A marked reduction in this behavior is observed on slippery floors, indeed [34]. According to different studies, an increase in activity during estrus indicated a reliable prediction of ovulation [35,36]. However, it is supposed that multiparous cows show a reduction in intensity and peak activity [36,37]. In fact, high milk production has been associated with negative effects in walking activity [37]. In addition, hot climatic conditions and heat stress are also associated with a reduction in activity during estrus [37,38]. During estrus, cows also show more antagonistic interactions and social interactions than during diestrus [39], but they can be negatively influenced by stocking density. In respect to pregnant cows, some of them might also show behavioral signs of estrus and, in this case, misinterpretation can lead to re-insemination of pregnant cows that can cause abortion [40,41,42]. 

## 5. Conclusions

Herein, we demonstrated that a MENT-EGAS prototype was capable distinguishing with correctness estrus from non-estrus phases in Holstein–Friesian cattle by direct sampling in the perineal region. Therefore, we provided for the first time evidence that an accurate, non-invasive method can be applied for estrus detection using an EN-based device. MENT-EGAS might be used in the future in milking parlors or milking robots as a routine device for detection of estrus. 

## Data Availability

The data that support the findings of this study are available from the corresponding author upon request.

## Supplementary Materials

The following supporting information can be downloaded at: https://www.mdpi.com/article/10.3390/vetsci9120688/s1, Figure S1: Portable electric nose version 3.5 (PEN 3.5).
