# Supplementary figures and images for: Estrus Detection in a Dairy Herd Using an Electronic Nose by Direct Sampling on the Perineal Region
Source: Vet Sci. 2022 Dec 9;9(12):688. doi: 10.3390/vetsci9120688 (PMC9786671; doi:10.3390/vetsci9120688)

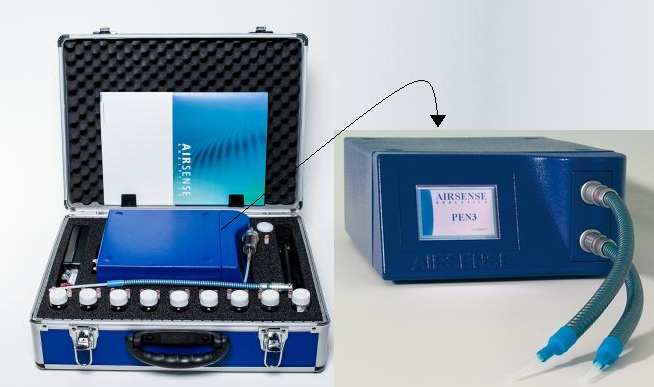

Supplement: Supplementary file 1 [file vetsci-09-00688-s001.zip › Figure S1-vetsci-1976293-supplementary.png]
